# Role of Leptin as a Link between Asthma and Obesity: A Systematic Review and Meta-Analysis

**DOI:** 10.3390/ijms24010546

**Published:** 2022-12-29

**Authors:** Helena Sánchez-Ortega, Carlos Jiménez-Cortegana, José P. Novalbos-Ruiz, Ana Gómez-Bastero, José G. Soto-Campos, Víctor Sánchez-Margalet

**Affiliations:** 1 Medicine Laboratory Service, Department of Medical Biochemistry and Molecular Biology and Immunology, Medical School, Virgen Macarena University Hospital, University of Seville, Av. Dr. Fedriani 3, 41009 Seville, Spain; 2 Department of Biomedicine, Biotechnology and Public Health, Medical School, University of Cadiz, 11003 Cádiz, Spain; 3 Pneumology Service, Virgen Macarena University Hospital, University of Seville, 41009 Seville, Spain; 4 Pneumology Service, Jerez University Hospital, University of Cadiz, 11003 Cádiz, Spain

**Keywords:** asthma, obesity, inflammation, adipokines, leptin, immune system

## Abstract

Asthma and obesity are considered as highly prevalent diseases with a great impact on public health. Obesity has been demonstrated to be an aggravating factor in the pathogenesis of asthma. Adipose tissue secretes proinflammatory cytokines and mediators, including leptin, which may promote the development and severity of asthma in obese patients. This study is a systematic review and a meta-analysis based on the relationship between leptin and asthma during obesity. MEDLINE, Cochrane, EMBASE and CINAHL databases were used. Data heterogeneity was analyzed using Cochran’s Q and treatment effect with the DerSimonian and Laird method. Random effect analyses were carried out to test data sensitivity. Asymmetry was estimated using Begg’s and Egger’s tests. All studies showed significant differences in leptin levels. The effect of the measures (*p* < 0.001), data sensitivity (*p* < 0.05) and data asymmetry were statistically significant, as well as tBegg’s test (*p* = 0.010) and Egge’s test (*p* < 0.001). Despite the existing limiting factors, the results of this study support the relevant role of leptin in the pathophysiology of asthma in obese subjects. Nevertheless, further studies are needed to obtain better insight in the relationship between leptin and asthma in obesity.

## 1. Background

Asthma, a chronic disorder characterized by inflammation, remodeling and the hyperresponsiveness of airways, provokes a progressive worsening of breathing, combined with coughing, tightness and/or wheezing. Asthma severity depends on the frequency and control of these exacerbations [1], which are measured by pulmonary function tests such as the force vital capacity (FVC) and the forced expiratory volume (FEV) [2]. Currently, asthma affects more than 300 million people worldwide, becoming a very prevalent disorder in our society [3]. Asthma has a genetic substrate and is highly influenced by external factors, including (but not limited to) exposure to allergens, pollutants, or bronchoconstriction after exercise, among others [4,5]. In all these settings, a pro-inflammatory state is ultimately promoted by the immune system, in which T-helper type 2 (Th2) cells express interleukin (IL)-4 and IL-13, and have been described as promoters of acute inflammation in the pathogenesis of asthma [6]. T cells also promote the activation of B cells, which, in turn, produce immunoglobulin (Ig) E, thus stimulating mast cells and producing the release of histamine [7].

All of these mechanisms induce hypersensitivity of the bronchial mucosa and the remodeling of the airway in response to prolonged inflammatory stimuli, making asthma a complex syndrome. Asthma could also become a more complex situation when it is associated with obesity, determined by a Body Mass Index (BMI) ≥ 30 kg/m^2^. This chronic disease is a well-known risk factor for asthma and is associated with both the severity and frequency of symptoms. Obesity also reduces the response to many medications and decreases the life quality of asthmatic patients [8]. Moreover, the inflammation promoted by adipocytes has been increased in obese subjects with asthma [9] due to the release of pro-inflammatory mediators, such as IL-6, TNF-a, or leptin, and the downregulation of anti-inflammatory factors, including adiponectin [10].

Specifically, leptin is a 16 kDa adipocyte-derived hormone not only expressed in adipose tissue, but also in other tissues and organs such as the stomach, skeletal muscle, pituitary cells, and the placenta, and has pleiotropic effects when bound to its receptor (Ob-R) [11]. Leptin is involved in metabolism and food intake [12], reproduction [13], immunometabolism [14], cancer [15], or non-alcoholic fatty liver disease [16], among many others. Leptin and its receptors share structure and functional similarities with the proinflammatory IL-6, and are able to activate different signaling pathways, including Janus Kinase (JAK)/Signal Transducer and Activator of Transcription (STAT), Phosphatidylinositol-3-Kinase (PI3K)/Protein Kinase B (Akt), and Mitogen Activated Protein Kinase (MAPK)/Extracellular-Signal-Regulated Kinase (ERK) cascades [17]. In immune cells, leptin induces allergic inflammatory responses by the proliferation and survival of type 2 innate lymphoid cells (ILC2) and Th2 cells [18]. Leptin also influences inflammatory responses by inducing the activation of monocytes and both CD4+ and CD8+ T cells [19,20], and the production of pro-inflammatory cytokines such as TNF-a or IL-18 [11], which may affect different respiratory diseases, including asthma [21].

The aim of this article was to evaluate the effects of circulating leptin in asthmatic patients with obesity by carrying out a systematic review and a meta-analysis in order to check whether some potential treatments (e.g., leptin inhibitors) may be useful in the future to ameliorate asthma symptoms in obese patients or even achieve a complete remission of the disease.

## 2. Material and Methods

### 2.1. Search Strategy

**Figure 1:**
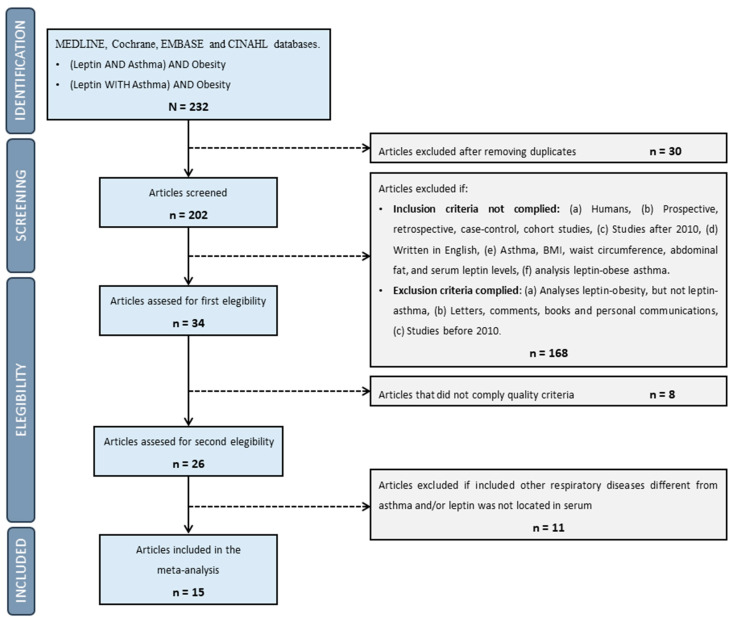
Study selection process for the meta-analysis review related to the role of leptin in asthma.

MEDLINE, Cochrane, EMBASE and CINAHL databases were used to search articles based on obese asthmatics and leptin. We carried out this meta-analysis using the following keywords: (leptin AND asthma) AND obesity, (leptin WITH asthma) AND obesity. In total, 232 searches were retrieved and reduced by using screening and eligibility methods, mainly inclusion/exclusion criteria and the evaluation of research quality, as shown in Figure 1.

### 2.2. Inclusion Criteria

The criteria for the inclusion of studies were as follows: (a) Studies based on human asthma, without considering other obstructive pathologies such as chronic obstructive pulmonary disease, (b) Prospective, retrospective, case-control, and cohort studies, (c) Published from 2010, (d) written in English, (e) Including the following variables: asthma, body mass index, waist circumference and/or abdominal fat, and serum leptin levels (ng/mL), (f) Analyses between leptin and obese patients with asthma.

### 2.3. Exclusion Criteria

The criteria for the exclusion of studies were as follows: (a) Studies that correlate obesity and asthma, with the absence of an analysis between leptin and asthma, (b) Letters, comments, books, and personal communications, (c) Studies published before 2010.

### 2.4. Quality Evaluation

The quality of the selected articles was evaluated according to the Newcastle–Ottawa Scale (NOS) for case-control and cohort studies [22] and the tool for the critical appraisal of epidemiological cross-sectional studies (CA-CSS) [23].

### 2.5. Data Extraction

Once the screening/eligibility process, shown in Figure 1, was completed, the following variables were extracted to describe the selected studies, as shown in Table 1: (a) author and year, (b) country, (c) study design, (d) quality scale, (e) types of subjects analyzed, (f) comparison groups in each study and number of subjects, (g) BMI (kg/m^2^) and (h) statistical measure for BMI since some studies used different statistical parameters. In Table 2, the following information was selected: (a) author and year, (b) comparison groups used in our analysis and (c) serum leptin levels (mean and standard deviation). In some studies, different statistical parameters were used, and leptin levels were recalculated to obtain mean, standard deviation and/or standard error.

### 2.6. Statistical Analysis

To check the comparability of data, heterogeneity and homogeneity were firstly analyzed by using Cochran’s Q test for 15 independent studies. As a result, analyzed articles were heterogeneous and random effect models were performed. The DerSimonian and Laird method was carried out due to its efficiency in estimating the treatment effect. All analyses were conducted by using Epidat v3.1 software (Galicia, Spain).

## 3. Results

### 3.1. Included Studies

A total of fifteen studies were eligible for inclusion in this meta-analysis (Figure 1), which included studies from USA (three studies), Turkey (two studies), Egypt (two studies) and France, Greece, Iran, Portugal, Mexico, South Korea, Canada, and Qatar (one study each one), as shown in Table 1. The studies comprised a total of 1933 subjects, including asthmatic and non-asthmatic individuals with and without obesity. Of them, we considered 964 obese patients with asthma or severe asthma (depending on the type of classification used in every research article) to be eligible for the meta-analysis.

### 3.2. Heterogeneity

**Figure 2:**
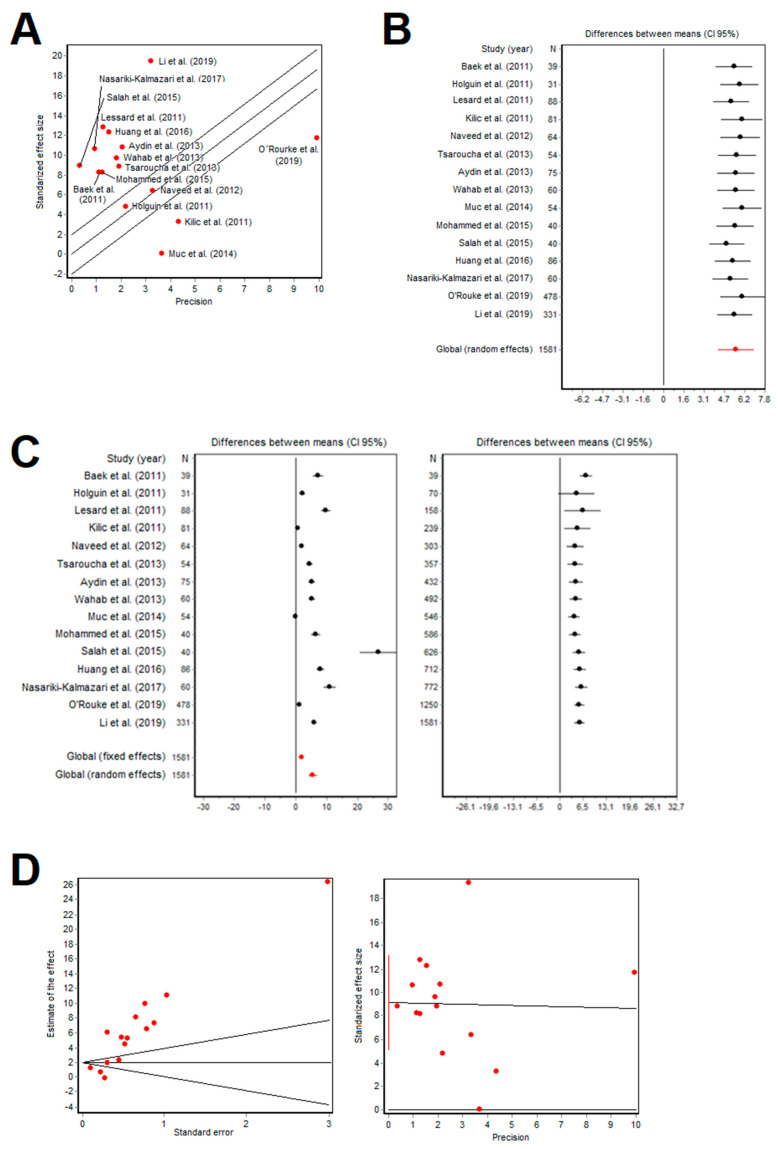
Results from the selected studies. (**A**): Galbraith graph to visualize data heterogeneity; (**B**): Forest chart to visualize robustness of leptin data without obesity; (**C**): Forest chart to visualize robustness of leptin data with obesity; (**D**): Funnel plot graph (left) and Egger’s regression plot (right) to visualize asymmetry [24,25,26,27,28,29,30,31,32,33,34,35,36,37,38].

The fifteen studies reported high levels of serum leptin in asthmatic patients with obesity, as shown in Table 2. All studies showed statistically significant differences in serum leptin levels between groups of individuals. Among studies, heterogeneity in leptin data was found, and the DerSimonian and Laird method was performed due to its efficiency in estimating the effect of the measures. The following results were obtained: Q = 823.63 and *p* < 0.001; coefficient of variation between studies was 1.34 and RI coefficient was 98.79%, which represent a high heterogeneity. Moreover, intra-study variance was 0.085, which supported the internal validity of the studies. A Galbraith graph was also made to visualize the heterogeneity (Figure 2A).

### 3.3. Sensitivity

#### 3.3.1. Leptin Data without BMI

The results of this analysis are found in the column *Random effect analysis for leptin levels* in Table 3. Differences between means and their CI (95%) were similar for each study, the global random effect obtained was 5.56 (4.17–6.96) ng/mL of serum leptin and *p* < 0.05, concluding the robustness of the results achieved, as illustrated in the Forest chart of Figure 2B.

#### 3.3.2. Leptin Data with BMI

The column *Fixed and random effect analysis for leptin levels and BMI* in Table 3 shows the variability in leptin levels between asthmatic patients with obesity and their controls. Global random effect analysis concluded in the same result for leptin levels: 5.56 (4.17–6.96) ng/mL and *p* < 0.05. A Forest chart for this analysis is presented in Figure 2C.

### 3.4. Asymmetry

Asymmetry of data was estimated by using both the Begg’s and Egger’s tests. Z value (Begg’s test) was 2.57 (*p* = 0.010) and T value (Egge’s test) was 4.95 (*p* < 0.001), which resulted in a publication bias and, consequently, the overestimation of the role of leptin in the analysis. To visualize the asymmetry, both a Funnel Plot graph and an Egger’s regression plot were illustrated in Figure 2D.

## 4. Discussion

Asthma and obesity are very prevalent diseases with a great global impact due to their high morbidity and mortality [39,40]. Although their relationship has been previously demonstrated [8,9], many other metabolic dysfunctions are also involved [41]. Adipose tissue has endocrine functions and promotes a cascade of pro-inflammatory cytokines and adipokines, including leptin, which may be a key factor in the pathology of asthma [27,37,38]; this is because the adipokine may induce changes in the mechanics and functions of the lungs via bronchial inflammation on admission, compared to the stable phase of the disease [42,43,44], mainly due to the accumulation of leptin-producing monocytes in the airway [45]. These ultimately favor the expansion in Th17 cells and the decrease in regulatory T cells [46]. In addition, leptin has been suggested to promote airway inflammation via upregulation of the mitochondrial reactive oxygen species/NOD-, LRR-, and the pyrin domain-containing protein 3 (mostly known as mtROS/NLRP3) inflammasome signaling pathway in human normal BEAS-2 bronchial epithelial cells in vitro [47]. The present work concluded in a systematic review and a meta-analysis to evaluate the potential relationship between the circulating leptin in obese patients and the severity of asthma. We finally analyzed fifteen studies that complied with the inclusion criteria. Most of them reported a stronger severity of asthma symptoms or higher exacerbations in obese patients, characterized by increased leptin levels and low adiponectin levels, compared with their non-obese counterparts. These results are supported by other studies [48,49], including the French EGEA study, which found that patients with severe asthma were characterized by high leptin levels, poor lung function, a chronic cough, high BMI, and high circulating neutrophil levels [50]. In addition, weight loss was associated with significant changes in the systemic and pulmonary inflammatory profiles in asthmatic patients, leading to a better control due to an increase in some anti-inflammatory mediators (e.g., adiponectin) and a reduction in pro-inflammatory mediators, including leptin [51].

Some of the studies included in this meta-analysis involved pulmonary function tests, such as an FEV in one second (FEV1) and FVC/FEV1 ratio, and their correlation with leptin levels or severity of asthma. Leptin was shown to be inversely correlated with both FEV1, FVC/FEV1 ratio [29,31,33,36], and FEV1/FVC ratio [35], which may suggest that nonatopic inflammation (including not only leptin, but also other adipokines such as adiponectin) increase the severity of asthma by obesity-dependent and independent mechanisms [52,53]. In addition, serum leptin levels were associated with maximum decreases in FEV1 after exercise [25], and increased the odds of an abnormal FEV1 [28]. However, other studies reported no association between obese and non-obese individuals with asthma, according to the percentage of FEV1 [24,26,30,34]. Similar results have been obtained in both asthmatic children and teenagers [25,35], rather than adult woman, probably because hormonal factors may be involved [26,31,32,36].

Generally, leptin levels are higher in women, and different leptin-associated pathologies have been more prevalent in the female population [54,55], including asthma [56], thus suggesting a positive association between hormonal changes caused by menopause, high leptin levels, and the severity of asthma. Interestingly, pregnant women with obesity with high cord blood leptin may have an increased risk of asthma [57]. However, Muc et al. (2014) reported no differences in leptin levels between asthmatic obese women and their non-asthmatic counterparts (78.12 ± 44.65 vs. 78.06 ± 54.65 ng/mL, respectively), but found significant differences when compared to asthmatic women with normal weight (39.66 ± 28.31 ng/mL; *p* = 0.006), suggesting that leptin levels were only BMI-dependent [32]. Moreover, Sutherland et al. (2009) showed high leptin levels in overweight and obese individuals, but no associations between leptin levels and a diagnosis of asthma, or some biomarkers, such as the bronchodilator response or FEV1/FVC ratio [58]. In children, a relationship between obesity and asthma has also been found via high leptin levels and a low adiponectin concentration in blood [59], suggesting that those adipokines, together with the BMI, may be potential predictive biomarkers for asthma [60].

Similarly, high serum leptin levels have been found, in asthmatic mice with obesity [61], to promote allergic airway inflammation in preclinical models. Interestingly, it has been shown that IL-33 needs leptin to induce airway inflammation and goblet cell metaplasia in obese mice [62], and leptin administration with allergens may increase serum IgE in mice [63]. In fact, leptin concentration has been decreased after using simvastatin in asthmatic mice with obesity [64], which reflects the importance of this adipokine in this pulmonary disorder. Moreover, it has been demonstrated that leptin has improved cytokine production by lung fibroblasts [65], and MUC5AC production by IL-13 in human bronchial epithelial cells [66], contributing to the worsening of asthma in obese individuals.

## 5. Conclusions

The results of this meta-analysis were largely limited by not only the lack of a consensus to measure serum leptin among studies, but also the different sample sizes used in every work, the different criteria used to classify the severity of asthmatic patients with obesity, as well as the type of control individuals recruited, which included non-obese patients with asthma and/or non-asthmatic subjects with different BMIs.

However, the results reveal the important role of leptin in the pathogenesis of asthma, suggesting that this adipokine may activate signaling pathways to promote both the inflammatory cascade and the parasympathetic system, which could negatively affect the bronchial tone, thus producing bronchoconstriction and bronchial hyperresponsiveness. In this sense, we strongly think that different preclinical studies should be performed to treat overweight or obese mice with asthma, which could be helpful to control symptoms or even achieve a complete remission of the disease. Some of these treatments could be therapies to target leptin or its receptors, and may also be tested in clinical trials for asthmatic patients with not only obesity, but also other leptin-related pathologies, including (but not limited to) cardiovascular diseases, diabetes mellitus, or rheumatoid arthritis, which have already been associated with asthma [67,68,69].

## Figures and Tables

**Table 1:** General characteristics of the fifteen studies analyzed. * There were no distinctions between groups to measure BMI. It also included other pathologies; ** This study did not measure BMI depending on asthma severity; *** This study did not specify BMI for asthma groups.

Study	Country	Study Design	Quality Scale	Types of Subjects	Comparison Groups (No of Patients)	BMI (kg/m^2^)	Statistical Measure for BMI
Holguin et al. (2011) [24]	USA	Cohorts	NOS 7/8	Adults	Obesity with asthma (14)	37 (34–42)	Median (IR)
					Overweight with asthma (2)	27 (26–28)	
					Lean with asthma (5)	22 (21–23)	
					Obesity w/o asthma (17)	33 (32–37)	
					Overweight w/o asthma (3)	28 (27–29)	
					Lean w/o asthma (7)	23 (22–24)	
Baek et al. (2011) [25]	South Korea	Cohorts	NOS 7/8	Children	Obesity with asthma (19)	23.0 ± 3.1	Mean ± SD
					Obesity w/o asthma (23)	23.3 ± 3.8	
					Normal weight with asthma (23)	16.9 ± 2.1	
					Healthy control (20)	16.8 ± 1.9	
Kilic et al. (2011) [26]	Turkey	Transversal	CA-CSS Medium	Adults	Obesity with asthma (41)	34.87 ± 4.26	Mean ± SD
					Non-obesity with asthma (40)	25.55 ± 2.84	
Lessard et al. (2011) [27]	Canada	Cohorts	NOS 7/8	Adults	Obesity with asthma (44)	37 ± 6	Mean ± SD
					Non-obesity with asthma (44)	23 ± 2	
Naveed et al. (2012) [28]	USA	Case-control	NOS 6/8	Adults	FEV ≥ LNN (218)	27.6 (26–30)	Median (IR)
					FEV < LNN (109)	29 (37–31)	
Aydin et al. (2013) [29]	Turkey	Cohorts	NOS 7/8	Adults	Asthmatics (45)	29.6 ± 5.4	Mean ± SD
					Non-asthmatics (30)	28.2 ± 5.3	
Wahab et al. (2013) [30]	Qatar	Cohorts	NOS 7/8	Children	Obesity with asthma (29)	N/A ***	-
					Non-obesity with asthma (31)		
Tsaroucha et al. (2013) [31]	Greece	Cohorts	NOS 8/8	Adults	Severe asthma (15)	36.5 ± 5.4	Mean ± SD
					Mild to moderate asthma (17)	32.3 ± 6.0	
					Control (22)	31.2 ± 6.1	
Muc et al. (2014) [32]	Portugal	Cohorts	NOS 8/8	Adults	Overweight with asthma (28)	30.4 ± 4.3	Mean ± SD
					Overweight w/o asthma (26)	28.9 ± 4.2	
					Normal weight with asthma (26)	21.6 ± 1.9	
Mohammed et al. (2015) [33]	Egypt	Cohorts	NOS 7/8	Adults	Obesity with asthma (20)	35.15 ± 3.32	Mean ± SD
					Obesity w/o asthma (8)	34.1 ± 1.2	
					Non-obesity with asthma (20)	23.15 ± 1.81	
					Healthy control (7)	23.7 ± 1	
Salah et al. (2015) [34]	Egypt	Cohorts	NOS 6/8	Adults	Obesity with asthma (20)	34.9 ± 2.4	Mean ± SD
					Obesity w/o asthma (20)	36.16 ± 3.15	
					Non-obesity with asthma (20)	22.97 ± 1.13	
					Healthy control (20)	22.9 ± 0.68	
Huang et al. (2016) [35]	Mexico	Cohorts	NOS 7/8	Teenagers	Obesity with asthma (28)	26.9 ± 3.0	Mean ± SD
					Obesity w/o asthma (46)	27.9 ± 3.2	
					Normal weight with asthma (58)	18.9 ± 3.2	
					Healthy control (63)	18.6 ± 2.0	
Nasiri-Kalmarzi et al. (2017) [36]	Iran	Transversal	CA-CSS High	Teenagers and adults	Severe asthma (25)	N/A **	-
					Moderate asthma (30)		
					Mild asthma (35)		
Li et al. (2019) [37]	France	Cohorts	NOS 8/8	Adults	Persistent asthma (305)	24.3 ± 4.3	Mean ± SD
					Remitted asthma (26)	22.6 ± 3.1	
O’Rourke et al. (2019) [38]	USA	Cohorts	NOS 8/8	Adults	No remitted asthma (89)	46.3 *	Median
					Remitted asthma (195)		

**Table 2:** Serum leptin data (ng/mL) used to perform the meta-analysis. * Data from the study were recalculated to obtain mean, standard deviation and/or standard error. ** This study did not calculate serum leptin levels for every group of patients.

Study	Comparison Groups	Leptin (ng/mL)		
		Mean	Standard Deviation	Standard Error *
Holguin et al. (2011) [24]	Obese with asthma	72.00 *	89.50 *	23.92
	Comparison group	34.00 *	38.00 *	9.22
Baek et al. (2011) [25]	Obese with asthma	14.14	7.35	1.69
	Comparison group	4.81	3.64	0.81
Kilic et al. (2011) [26]	Obese with asthma	22.60 *	52.40 *	8.18
	Comparison group	16.70 *	47.21 *	7.46
Lessard et al. (2011) [27]	Obese with asthma	57.70	30.60	4.61
	Comparison group	19.50	19.50	2.94
Naveed et al. (2012) [28]	Obese with asthma	8.04 *	8.88 *	1.62
	Comparison group	5.37 *	6.48 *	1.11
Aydin et al. (2013) [29]	Obese with asthma	70.18	30.47	4.54
	Comparison group	34.38	51.19	9.35
Wahab et al. (2013) [30]	Obese with asthma	25.80	11.10	5.03
	Comparison group	8.80	11.10	1.99
Tsaroucha et al. (2013) [31]	Obese with asthma	24.80	14.80	2.62
	Comparison group	13.70	10.00	2.13
Muc et al. (2014) [32]	Obese with asthma	78.12	44.65	8.44
	Comparison group	78.06	54.65	10.72
Mohammed et al. (2015) [33]	Obese with asthma	92.90	8.00	1.79
	Comparison group	80.40	9.20	2.06
Salah et al. (2015) [34]	Obese with asthma	39.74	3.26	0.73
	Comparison group	23.58	1.99	0.44
Huang et al. (2016) [35]	Obese with asthma	49.20	27.70	5.23
	Comparison group	20.00	18.90	2.48
Nasiri-Kalmarzi et al. (2017) [36]	Obese with asthma	50.60	19.20	3.84
	Comparison group	20.40	9.40	1.59
Li et al. (2019) [37]	Obese with asthma	4.40	3.60 *	0.21
	Comparison group	3.00	2.20 *	0.43
O’Rourke et al. (2019) [38]	Obese with asthma	56.80 *^,^**	58.10 *	3.45
	Comparison group			4.17

**Table 3:** Results from random effect analysis for leptin levels, and from fixed and random effect analysis for leptin levels and BMI.

	Random Effect Analysis for Leptin Levels			Fixed and Random Effect Analysis for Leptin Levels and BMI			
	N	D (CI 95%)	Relative Change	N	D (CI 95%)	Weight %	
						Fixed Effects	Random Effects
Holguin et al. (2011) [24]	1550	5.85 (4.36–7.33)	5.09	31	2.18 (1.29–3.07)	2.7367	7.0404
Baek et al. (2011) [25]	1542	5.43 (4.01–6.85)	−2.44	39	7.31 (5.57–9.04)	0.7201	6.5136
Kilic et al. (2011) [26]	1500	6.02 (4.44–7.61)	8.28	81	0.75 (0.30–1.20)	10.7126	7.1951
Lessard et al. (2011) [27]	1493	5.20 (3.83–6.56)	−6.56	88	9.88 (8.36–11.39)	0.9449	6.6749
Naveed et al. (2012) [28]	1517	5.90 (4.37–7.44)	6.11	64	1.94 (1.35–2.54)	6.1471	7.1550
Aydin et al. (2013) [29]	1506	5.59 (4.15–7.03)	0.41	75	5.21 (4.26–6.17)	2.3924	7.0112
Wahab et al. (2013) [30]	1521	5.59 (4.15–7.03)	0.43	60	5.21 (4.15–6.27)	1.9324	6.9568
Tsaroucha et al. (2013) [31]	1527	5.64 (4.19–7.10)	1.46	54	4.56 (3.55–5.58)	2.1021	6.9795
Muc et al. (2014) [32]	1527	6.03 (4.53–7.54)	8.47	54	0.01 (−0.53–0.54)	7.6383	7.1733
Mohammed et al. (2015) [33]	1541	5.49 (4.06–6.91)	−1.37	40	6.48 (4.93–8.03)	0.9057	6.6520
Salah et al. (2015) [34]	1541	4.86 (3.51–6.22)	−12.57	40	26.76 (20.86–32.66)	0.0626	3.1520
Huang et al. (2016) [35]	1495	5.34 (3.95–6.72)	−4.10	86	8.10 (6.81–9.39)	1.3035	6.8237
Nasiri-Kalmazari et al. (2017) [36]	1521	5.16 (3.78–6.54)	−7.22	60	10.97 (8.94–12.99)	0.5290	6.2831
Li et al. (2019) [37]	1250	5.46 (4.10–6.82)	−1.84	331	6.06 (5.45–6.67)	5.8304	7.1499
O´Rourke et al. (2019) [38]	1103	6.07 (4.36–7.78)	9.07	478	1.17 (0.97–1.37)	56.0423	7.2392
**Global**	**1581**	**5.56 (4.17–6.96)**	-	-	-	-	-
**Fixed effects**	-	-	-	1581	1.98 (1.83–2.12)	-	-
**Random effects**	-	-	-	**1581**	**5.56 (4.17–6.96)**	-	-

## Data Availability

The datasets during and/or analyzed during the current study are available from the corresponding author on reasonable request.

## Abbreviations

BMI	Body mass index.
CA-CSS	Critical appraisal of epidemiological cross-sectional studies.
FEV	Forced expiratory volume.
FVC	Force vital capacity.
Ig	immunoglobulin.
IL	Interleukin.
NOS	Newcastle–Ottawa Scale.
Ob-R	Leptin receptor.
TNF-a	Tumor necrosis factor Alpha.
